# Genetics and Pathogenicity of Natural Reassortant of Infectious Bursal Disease Virus Emerging in Latvia

**DOI:** 10.3390/pathogens11101081

**Published:** 2022-09-22

**Authors:** Anna Pikuła, Anna Lisowska

**Affiliations:** Department of Poultry Diseases, National Veterinary Research Institute, Al. Partyzantow 57, 24-100 Pulawy, Poland

**Keywords:** infectious bursal disease virus, reassortment, viral evolution, virulence

## Abstract

Infectious bursal disease virus is an immunosuppressive pathogen that, despite applied vaccination, is affecting the poultry industry worldwide. This report presents the genetic and pathotypic characterization of a natural reassortant emerging in Europe (Latvia). Genetic characterization showed that strain 25/11/Latvia/2011 represents genotype A3B1, whose segment A is derived from very virulent strains, while segment B is from the classic-like genogroup. Phylogenetic maximum likelihood inference of the B-segment sequence clustered the reassortant strain together with the US antigenic variant E strain. However, the obtained full-length sequence of 25/11/Latvia/2011 revealed that not only reassortment but also dozens of mutations shaped the unique genetic makeup. Phenotypic characterization showed no mortality and no clinical signs of disease but a severe bursa of Fabricius atrophy and splenomegaly in the convalescent birds at 10 days post infection. The results obtained indicate that the acquired genetic constellation contributed to a decrease in virulence; nevertheless, the infection causes severe damage to lymphoid organs, which can lead to impaired immune responses.

## 1. Introduction

Infectious bursal disease virus (IBDV), a member of the *Birnaviridae* family, *Avibirnavirus* genus, is a global pathogen that leads to an immunosuppressive disorder known as Gumboro disease (IBD). The virus has a bisegmented double-stranded RNA genome [1] that encodes five viral proteins. Segment A contains information about the structure of four proteins (VP2-VP5), including the VP2 capsid protein, which is responsible for antigenicity and tissue tropism [2]. The monocistronic segment B encodes viral polymerase (RdRp, VP1) that manages replication, transcription and translation [3,4]. Using reverse genetics, it has been shown that both segments affect virus virulence [5]; however, the precise mechanism of pathogenicity is still unknown. IBDVs can be differentiated based on antigenicity, virulence or genetic composition; although genotype should not be strictly combined with antigenicity or pathotype, they often overlap [6]. Thus, point genome mutations within an antigenic epitope can change the antigenicity of IBDV without significantly changing its position on the phylogenetic tree [7]. Similarly, a mutation in the genome at a virulence-relevant site can alter the pathotype of a virus without changing its genotype [5]. 

The current global epidemiological situation is shaped by both low-to-moderate [8,9,10,11,12] and highly pathogenic strains [13,14]. In the last decade, a shift has been observed in the population of IBDV genetic lineages circulating in Europe, previously dominated by very virulent strains. Molecular surveys confirmed the emergence of three types of natural reassortants representing genotypes A3B4, A3B1 and A9B1. The first mentioned are prevalent in Poland [12], but their presence has also been confirmed in Finland [15]. Strains of the A3B4 genotype show a pathotype comparable to classical virulent strains, and the infection of SPF chickens resulted in 20-30% mortality [12]. In turn, genotype A3B1 has been confirmed in many European countries including the Netherlands, Belgium, Denmark, Germany, the Czech Republic and Sweden [10]. These strains are less virulent compared to very virulent IBDV, and no mortality or clinical signs were observed in the experimental infection; however, marked atrophy of the bursa of Fabricius indicates major immunosuppressive implications. Moreover, a new genotype A9B1 of IBDV was recently identified in Portugal, but the pathogenicity of the virus has not yet been determined [16].

The presented study reports the genetic and pathotypic characteristics of an IBDV reassortant detected in Latvia, belonging to the A3B1 genotype. Interestingly, full-sequence analysis of the B segment did not confirm phylogenetic relatedness to classical attenuated strains, as in previously reported reassortants in Europe [10], indicating the circulation of more IBDV mosaic groups. 

## 2. Results

### 2.1. Molecular and Phylogenetic Analysis

**Figure 1:**
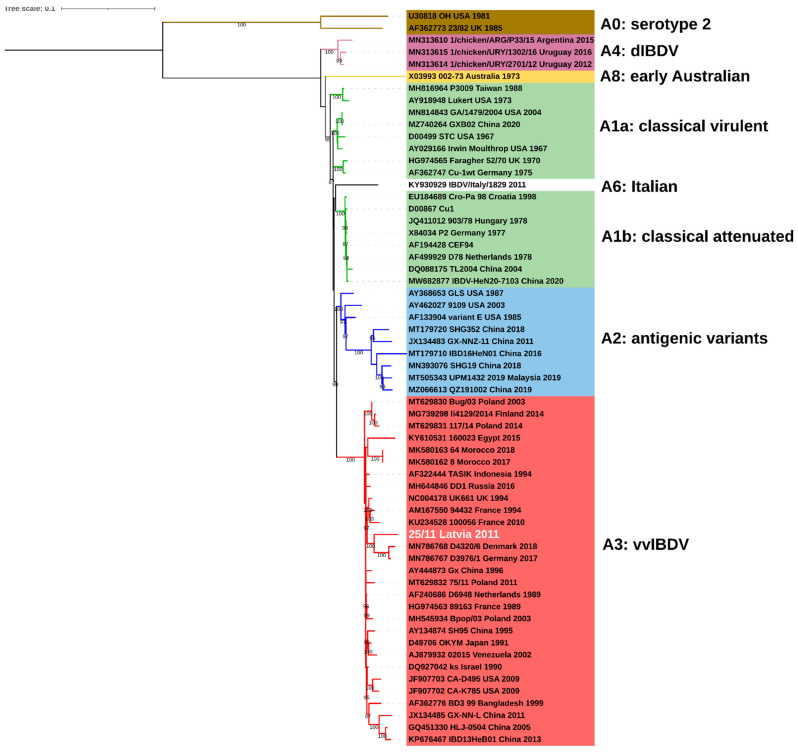
Phylogenetic tree based on full coding sequence of IBDV for viral segment A (3076nt) using 60 reference strains retrieved from GenBank and maximum likelihood (ML) method, GTR+F+I+G4 model. The main IBDV genogroups according to classification of Islam et al. [18] are denoted (**A1**: green, **A2**: blue, **A3**: red, **A4**: purple, **A6**: white, **A8**: yellow, **A0**: brown).

**Figure 2:**
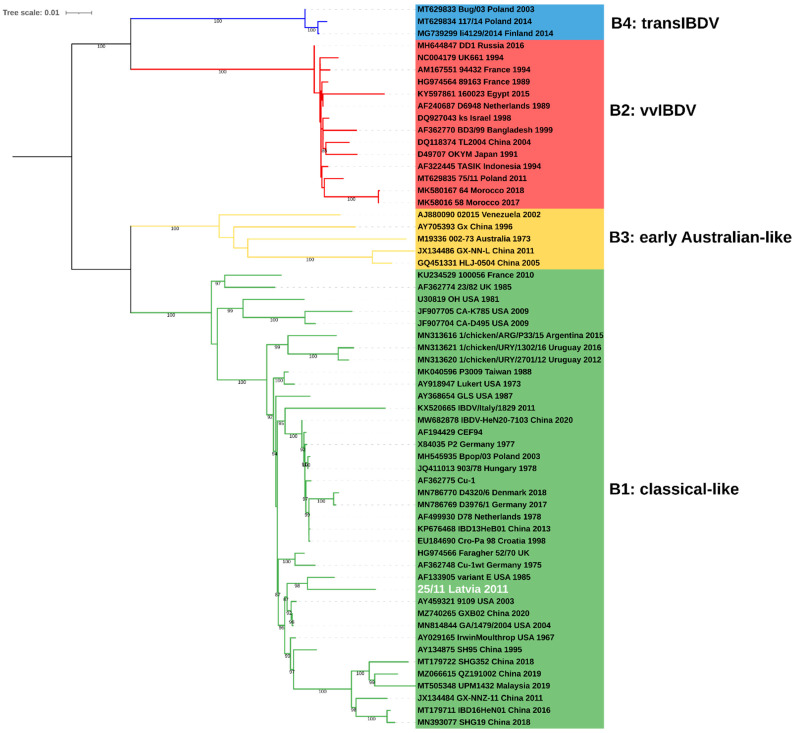
Phylogenetic tree based on full coding sequence of IBDV for viral segment B (2640nt) using 59 reference strains retrieved from GenBank and maximum likelihood (ML) method, GTR+F+I+G4 model. The main IBDV genogroups according to classification of Islam et al. [18] are denoted (**B1**: green, **B2**: red, **B3**: yellow, **B4**: blue).

The obtained complete consensus sequence of 25/11/Latvia/2011 of both segments was deposited in the GenBank database under accession numbers OP225970 and OP225971. The maximum likelihood (ML) phylogenetic inference based on the full coding sequence confirmed that IBDV strain 25/11/Latvia/2011 is a reassortant whose segment A is derived from very virulent strains (genogroup A3) (Figure 1), while segment B represents the B1 genogroup (Figure 2). The B1 group is the most abundant and includes classical (virulent and attenuated), US antigenic variant, distinct (dIBDV), Italian atypical and serotype 2 strains, as indicated by the calculated nucleotide identity of all full-length sequences deposited in GenBank, which ranges from 92.3 to 100%. However, the 25/11/Latvia/2011 strain forms a monophyletic branch together with the US and Chinese antigenic variants and classical virulent IBDVs (Figure 2). The highest nucleotide similarity for full-length segment A was found with very virulent IBDV strain D6948 (AF240686), which was 97.0%. In contrast, for segment B, 97.0% and 96,9% identity was found with strains GA/1479/2004 (MN814844, classical virulent) and variant E (AF133905, US antigenic variant), respectively. The demonstrated 3% difference in nucleotide similarity for the full sequences of the two segments shows that there are no closely related IBDV strains in the GenBank database. In contrast, partial sequence analysis of VP2 and VP1 showed high nucleotide similarity, 99.5-99.7% and 99.4-99.5%, respectively, with previously reported IBDV strains from Latvia [17]. Amino acid analysis of deduced viral proteins revealed the presence of 18 and 7 altered residues within proteins encoded by segments A and B, compared to strains D6948 and GA/1479/2004 (Table 1). Some of these alterations are unique to the characterized strain, including ^5^A, ^47^T and ^134^N in nonstructural VP5; ^545^T, ^570^V and ^680^F in viral protease VP4; ^935^V and ^960^D in structural VP3 protein; and ^71^D, ^157^L, ^455^S and ^759^R in viral polymerase (VP1).

### 2.2. Animal Experimental Study

**Figure 3:**
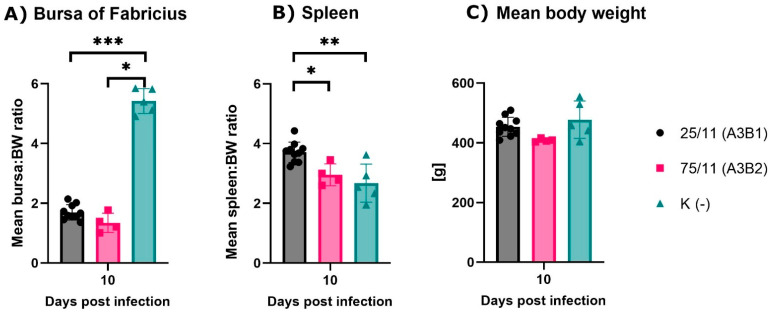
Mean bursa (**A**) and spleen (**B**) to body weight (BW) ratios and mean body weight of experimental groups. Each bar indicates the mean ± SD and statistical significance (* *p* < 0.05, ** *p* < 0.01, *** *p* < 0.001).

During observation, no mortality or clinical symptoms of the disease were observed in the normal group and in birds infected with the reassortant 25/11/Latvia/2011 (A3B1), while in the very virulent (A3B2) group, birds showed clinical signs such as ruffling of the feather, depression, diarrhea and a 60% mortality rate. At 10 dpi, severe bursa atrophy and splenic enlargement were observed at necropsy in both infected groups, as confirmed by the calculated bursa and spleen development ratios (Figure 3A). At 10 dpi, both groups had significantly lower bursa to body weight (B-BW) ratios (G1 *p* < 0.005, G2 *p* < 0.05) compared to the uninfected control group. In contrast, the calculated spleen to body weight (S-BW) ratio was significantly higher only in the reassortant group compared to both the control (*p* < 0.01) and very virulent groups (*p* < 0.05) (Figure 3B). Analysis of the mean body weight showed no significant differences in birds after recovery, and the results are shown in Figure 3C. The histopathological examinations of bursal tissue collected from euthanized birds at 10 dpi from both infected groups showed severe lymphoid necrosis due to follicle and lymphocyte depletion and hyperplasia of interfollicular fibrous connective tissue with moderate lymphoid and heterophilic infiltration. 

## 3. Discussion

The reassortment phenomenon is a common evolutionary mechanism among RNA viruses with a segmented genome that can lead to a large shift in phenotype, including virulence and transmission efficiency, thus posing a threat to public and animal health [19,20]. As retrospective studies have shown, the IBDV reassortment event was the genesis of a worldwide epidemic of acute Gumboro disease caused by the emergence of very virulent strains [21]. The implementation of routine immunoprophylaxis and the maintenance of biosecurity rules have reduced direct losses in poultry production, but the virus still causes economic losses due to immunosuppression. Thus, continuous field monitoring of important pathogens is key to maintaining the status quo.

Recently, IBDV reassortants of genotype A3B1 were reported in North-West Europe [10,17]. Genetic constellation of these mosaic strains exhibited that they arose from the exchange of segments A and B from very virulent and classical vaccine strains, respectively. As shown by in vivo studies, these strains do not cause mortality and clinical signs in infected SPF chickens but cause severe immunosuppression [10]. Presented genetic studies confirmed that strain 25/11/Latvia/2011 also represents A3B1 genotype reassortants, but the B segment is not vaccine-like but more closely related to classical virulent strains or US antigenic variants, indicating more recombinant virus types in Europe. To date, a similar IBDV reassortant has been detected in China [22]; the SH95 strain also showed the highest similarity of segment B to the variant E strain and segment A to the very virulent strains. Nonetheless, phylogenetic analysis revealed distinct origins of the Latvian and Chinese strains (Figure 2), which was supported by the relatively distant nucleotide identity of 96.4% and 95.9% for the A and B segments, respectively.

The obtained full-length sequence of 25/11/Latvia/2011 revealed that not only reassortment but also mutations shaped the unique genetic makeup. A total of 18 residue changes were found within proteins encoded by segment A compared to the closest reference strain D6948 (Table 1). As many as six amino acid changes were found within the non-structural VP5 protein, which is responsible for the release of virus progeny [23] by regulating the process of apoptosis [24,25] of the infected cell. Two alterations were found within the polycationic C-terminal region (residues 132–143), which is essential for targeting VP5 in the cell plasma membrane and efficient virus dissemination and in the transmembrane domain within the central region of the polypeptide (residues 69–88) [26]. However, the very limited information available on VP5 makes it difficult to conclude whether the changes shown may have affected the function of the protein. Among the changes found in the capsid protein, three are located within the antigenic domains (^219^L, ^220^F and ^254^D), which may contribute to antigenic drift rendering the vaccine less effective [27]. In contrast, the demonstrated replacement of aspartic acid to asparagine at position 279 of the VP2 protein was observed during attenuation of the very virulent OKYM strain [28]. All detected non-synonymous mutations within VP3 were located at the C-terminal part (Table 1). This multifunctional structural protein plays a role as a scaffold for the capsid [29] and viral replication [30]. Using reverse genetics, it was shown that the replacement of the C-terminal part of VP3 of the vvIBDV strain with the corresponding part of the serotype 2 isolate resulted in reduced virulence in SPF chickens [31]. Interestingly, the swapped VP3 fragment differed by six amino acids, and one of them, proline, at position 906 is present in the 25/11/Latvia/2011 strain. Another indicated amino acid substitution, alanine, to valine at position 990 reduced viral replication of the Gt strain [32]. Also within VP4, a serine protease, three altered residues were found; however, no link between these changes and the function of the enzyme was demonstrated so far. The VP1 protein, which is a viral polymerase encoded by segment B, has a number of amino acid changes typical of strains belonging to genogroup B1 (Table 1); in addition, five rare residue replacements were found, including the presence of proline at position 687 typically found in vvIBDV and the B3 genogroup. As indicated, strain 25/11/Latvia/2011 acquired a number of altered amino acids that may affect IBDV antigenicity and replication, or lead to attenuation, but only further study employing reverse genetics will demonstrate the impact of the listed changes. Interestingly, an unusually high number of altered amino acid residues were also observed in the 25/11/Latvia/2011 sequence compared to IBDV found in Europe representing the A3B1 [10] and A3B4 genotypes [12]. It is difficult to determine whether this is the result of an accumulation of mutations or whether we are dealing with the circulation of a distinct genetic lineage of the virus, previously undetected. High nucleotide similarity to Latvian strains (D1526/2/1/10LV, D2323/1/13LV) for VP2 and VP1 partial sequences indicates that this IBDV genetic lineage circulated in Latvia between 2010 and 2013, demonstrating the fitness of the resulting genetic constellation.

Animal trials showed a decrease in the virulence of the tested A3B1 genotype reassortant compared to the very virulent A3B2 strain. Infection with the 25/11/Latvia/2011 strain did not cause mortality or disease symptoms. In contrast, the observed severe lymphoid necrotic lesions in the bursa of Fabricius leading to significant atrophy and splenomegaly at day 10 post infection may indicate that infection can affect the function of these organs, especially the impairment of the host immune response [33]; nevertheless, further studies should be conducted to demonstrate their importance in this process. Loss of IBDV strain virulence resulting from reassortment has been frequently reported, specifically when a very virulent A segment is combined with a B segment of various origins, including vaccine-like (B1 genogroup) [10,34], serotype 2 (B1) [35], early Australian-like (B3) [36] or transitional IBDV (B4) [12]. In contrast, viruses belonging to the A3B1 genotype have also been reported to retain virulence, causing high mortality rates, such as the reassortants detected in Algeria [37] and South Korea [38]. Escaffre et al. (2013) indicated that both genome segments contribute to the virulence of very virulent strains [5], but given the lack of knowledge regarding the precise mechanisms of IBDV virulence, it cannot be ruled out that both the acquired B segment and the observed changes in viral proteins affect the pathogenicity of the detected strain. Nevertheless, only undertaking studies using reverse genetics would clarify the contribution of particular changes in the strain’s genome to the virulence process. Unfortunately, the pathogenicity of SH95, the only strain with a similar genetic composition, has not been studied.

Epidemiological data on the incidence of Gumboro disease show that IBDV reassortants are increasingly being isolated from field cases [10,39,40,41], indicating that the virus is continuously evolving, seeking new characteristics to facilitate its persistence in the environment. Strain 25/11/Latvia/2011 is a good example of this trend; its reduced virulence results in a subclinical course of the disease. Detection of such viruses is more difficult because the observed symptoms are nonspecific or less visible, which promotes the spread of the virus in the field. Thus, it is extremely important to conduct continuous field monitoring of IBDV using molecular methods based on both segments and to tighten biosecurity standards in case of field strain emerges. From a practical point of view, it would also be very important to determine whether the identified amino acid changes within the antigenic epitopes can affect the effectiveness of the vaccines used.

## 4. Materials and Methods

### 4.1. Full-length Genome Sequencing

In the present study, bursal tissue from a reported case of Gumboro disease [42] was used and prepared for high-throughput sequencing as previously described [43]. Sequencing was performed using the MiSeq System (Ilumina, San Diego, CA, USA) with 2 × 250 paired-end reads by commercial service (Genomed, Warsaw, Poland). CLC Genomics Workbench v7.0.4 was used for all bioinformatics analyses. 

### 4.2. Phylogenetic Analysis 

Sixty full coding sequences of IBDV reference strains downloaded from GenBank were included in the analyses (Table 2). Sequences of recent A3B1 European reassortants were also used for partial VP2 and VP1 analysis. The alignment of all sequence sets was performed using ClustalW method implemented in MEGAX program [44]. IQ-TREE software (version 1.6.12) was used both to estimate the best evolutionary model and to infer phylogenetic trees by maximum likelihood algorithm, and confidence levels for the branches were determined by Shimodaira–Hasegawa test and 1000 replicates of bootstrap [45]. The nucleotide identity was assessed using Geneious Prime 2022.1.1. The tree visualization was performed using the iTOL v6 online tool [46].

### 4.3. Animal Experimental Study

Animal experiments were carried out in accordance with the requirements and authorization of the local ethical commission (Permit no. 79/2015). A total of 25 five-week-old SPF chickens (VALO BioMedia, Osterholz-Scharmbeck, Germany) were randomly divided into 3 experimental groups: 10 birds in groups 1 and 2 and 5 birds in group 3. Chickens of groups 1 (G1) and 2 (G2) were intraocularly inoculated with 10^5^ EID_50_/0,1ml of the reassortant 25/11/Latvia/2011 (A3B1) and very virulent IBDV (A3B2) reference strain 75/11/Poland/2011, respectively, whereas birds from group 3 served as normal control and were inoculated with the same volume of phosphate-buffered saline. Chickens from all groups were separately housed in HEPA-filtered isolators (Montair Andersen B.V., Kronenberg, Netherlands) with access to water and feed ad libitum and monitored daily for 10 days for clinical symptoms. At the end of the experiment, all surviving birds were euthanized, and bursas of Fabricius and spleens were taken for further investigations. At 10 dpi, the euthanized birds, bursas and spleens were weighed for calculation of ratios of bursa and spleen to body weight according to previously published formula [47]. Half of each bursa was fixed in 10% phosphate-buffered formalin for HE staining and histopathological examination as previously described [43].

### 4.4. Statistical Analysis

The obtained data were analyzed using Graphpad Prism software 9.4.1 (Graphpad Prism Software Inc., San Diego, CA, USA). The mean with SD of ratios of bursa or spleen to body weight (B-BW and S-BW) and mean body weight calculated for each bird from the experimental groups were compared using the Mann–Whitney test. The results with *p* < 0.05 were considered statistically significant.

## Figures and Tables

**Table 1:** Amino acid differences in viral proteins encoded by both genome segments between 25/11/Latvia/2011 and IBDV representatives of different genogroups.

**Segment A**																											
			**VP5**								**VP2**									**VP4**				**VP3**			
**IBDV Strain**	**Genogroup**		**9**	**14**	**19**	**45**	**47**	**74**	**112**	**134**	**219**	**220**	**222**	**242**	**254**	**256**	**279**	**280**	**284**	**545**	**553**	**570**	**680**	**905**	**935**	**960**	**990**
D6948	A3	vv	D	E	N	R	A	F	A	H	Q	Y	A	I	G	I	D	N	A	A	R	M	Y	L	A	E	A
25/11	A3	vv	A	K	.	.	T	L	V	N	L	F	.	.	D	.	N	T	.	T	.	V	F	P	V	D	V
D3976	A3	vv	-	K	D	.	.	L	V	.	L	.	.	.	D	.	N	T	.	.	.	.	.	.	.	.	V
D4320	A3	vv	-	K	D	.	.	L	V	.	L	.	.	.	D	.	N	T	.	.	K	.	.	.	.	.	V
Bpop/03	A3	vv	.	.	.	.	.	.	.	.	.	.	.	.	.	.	.	.	.	.	.	.	.	.	.	.	.
SH95	A3	vv	-	.	.	.	.	.	.	.	.	.	.	.	.	.	.	.	.	.	.	.	.	.	.	.	V
GA/1479/2004	A1	cv	.	K	.	G	.	I	.	.	.	.	P	V	.	V	.	.	.	.	.	.	C	.	.	.	.
Variant E	A2	av	.	K	.	G	.	I	.	.	.	.	T	V	S	V	N	.	.	.	.	.	C	.	.	.	.
117/04	A3	vv	.	K	.	.	.	.	.	.	.	.	.	.	.	.	.	.	.	.	.	.	.	.	.	.	V
02015.1	A3	vv	.	.	.	.	.	.	.	.	.	.	.	.	.	.	.	.	.	.	.	.	.	.	.	.	V
HLJ-0504	A3	vv	.	K	.	.	.	.	.	N	.	.	.	.	.	.	.	.	.	.	.	.	.	.	.	.	.
100056	A3	vv	.	K	.	.	.	L	.	.	.	.	.	.	S	.	.	.	.	.	.	.	.	P	.	.	V
160021	A3	vv	.	K	.	.	.	.	.	.	.	F	.	.	S	.	.	.	.	.	.	.	.	.	.	.	.
**Segment B**																											
			**VP1**																								
**IBDV Strain**	**Genogroup**		**4**	**13**	**61**	**71**	**145**	**146**	**147**	**157**	**242**	**275**	**287**	**390**	**393**	**455**	**508**	**511**	**515**	**562**	**646**	**682**	**685**	**687**	**695**	**759**	**859**
GA/1479/2004	B1	cl	I	K	V	E	N	E	D	Q	D	I	T	L	E	T	R	R	D	S	G	R	V	S	K	K	T
25/11	B1	cl	.	.	.	D	.	.	.	L	.	.	.	.	.	S	.	.	.	.	.	.	I	P	.	R	.
D3976	B1	cl	.	T	.	.	.	.	G	.	.	L	.	.	.	.	.	K	.	.	.	K	.	.	.	.	V
D4320	B1	cl	.	T	.	.	.	.	G	.	.	L	.	.	.	.	.	K	.	.	.	K	.	.	.	.	V
Bpop/03	B1	cl	.	T	.	.	.	.	G	.	.	.	.	.	.	.	.	.	E	.	.	K	.	.	.	.	.
SH95	B1	cl	.	T	.	.	.	.	.	.	.	.	.	.	.	.	K	.	.	.	.	K	.	.	.	.	I
Variant E	B1	cl	.	.	.	.	.	.	G	.	.	.	.	.	.	.	.	.	.	.	.	.	I	.	.	.	.
117/04	B4	trans	V	.	I	.	S	.	G	.	.	.	A	M	.	.	K	S	E	.	S	K	.	.	.	.	I
02015.1	B3	eA	V	.	.	.	T	.	S	.	.	.	A	.	D	.	K	S	E	.	S	K	.	P	.	.	I
HLJ-0504	B3	eA	V	.	I	.	T	.	G	.	.	.	A	.	.	.	K	S	E	.	S	K	.	P	.	.	.
100056	B1	cl	.	.	.	.	.	.	G	.	.	.	.	.	.	.	K	S	E	A	.	K	.	.	.	.	.
160021	B2	vv	.	.	I	.	T	D	N	.	E	.	A	M	D	.	K	S	E	P	S	K	.	P	R	.	.
D6948	B2	vv	V	.	I	.	T	D	N	.	E	.	A	M	D	.	K	S	E	P	S	K	.	P	R	.	.

vv, very virulent; cv, classical virulent; av, US antigenic variant; cl, classical-like; trans, transIBDV; eA, early Australian-like. Dots show aa identity with reference strain D6948 and GA/1479/2004 for segments A and B, respectively.

**Table 2:** List of IBDV reference strains used for genetic analysis.

No.	IBDV Strain	Genotype	Origin		Segment A	Segment B
			Country	Date		
1	002-73	A8B3	Australia	1973	X03993	M19336
2	02015.1	A3B3	Venezuela	2002	AJ879932	AJ880090
3	1/chicken/ARG/P33/15	A4B1	Argentina	2015	MN313610	MN313616
4	1/chicken/URY/1302/16	A4B1	Uruguay	2016	MN313615	MN313621
5	1/chicken/URY/2701/12	A4B1	Uruguay	2012	MN313614	MN313620
6	100056	A3B1	France	2010	KU234528	KU234529
7	117/14/Poland/2014	A3B4	Poland	2014	MT629831	MT629834
8	160023	A3B2	Egypt	2015	KY610531	KY597861
9	23/82	A0B1	UK	1985	AF362773	AF362774
10	64	A3B2	Morocco	2018	MK580163	MK580167
11	75/11/Poland/2011	A3B2	Poland	2011	MT629832	MT629835
12	8	A3B2	Morocco	2017	MK580162	MK580165
13	89163	A3B2	France	1989	HG974563	HG974564
14	903/78	A1bB1	Hungary	1978	JQ411012	JQ411013
15	9109	A2B1	USA	2003	AY462027	AY459321
16	94432	A3B2	France	1994	AM167550	AM167551
17	BD3/99	A3B2	Bangladesh	1999	AF362776	AF362770
18	Bpop/03/Poland/2003	A3B1	Poland	2003	MH545934	MH545935
19	Bug/03/Poland/2003	A3B4	Poland	2003	MT629830	MT629833
20	CA-D495	A3B1	USA	2009	JF907703	JF907704
21	CA-K785	A3B1	USA	2009	JF907702	JF907705
22	CEF94	A1bB1	-	-	AF194428	AF194429
23	Cro-Pa/98	A1bB1	Croatia	1998	EU184689	EU184690
24	Cu-1	A1bB1	vaccine	-	D00867	AF362775
25	Cu-1wt	A1aB1	Germany	1975	AF362747	AF362748
26	D3976/1	A3B1	Germany	2017	MN786767	MN786769
27	D4320/6	A3B1	Denmark	2018	MN786768	MN786770
28	D6948	A3B2	Netherlands	1989	AF240686	AF240687
29	D78	A1bB1	Netherlands	1978	AF499929	AF499930
30	DD1	A3B2	Russia	2016	MH644846	MH644847
31	Faragher 52/70	A1aB1	UK	1970	HG974565	HG974566
32	GA/1479/2004	A1aB1	USA	2004	MN814843	MN814844
33	GLS	A2B1	USA	1987	AY368653	AY368654
34	Gx	A3B3	China	1996	AY444873	AY705393
35	GXB02	A1aB1	China	2020	MZ740264	MZ740265
36	GX-NN-L	A3B3	China	2011	JX134485	JX134486
37	GX-NNZ-11	A2B1	China	2011	JX134483	JX134484
38	HLJ-0504	A3B3	China	2005	GQ451330	GQ451331
39	IBD13HeB01	A3B1	China	2013	KP676467	KP676468
40	IBD16HeN01	A2B1	China	2016	MT179710	MT179711
41	IBDV/Italy/1829/2011	A6B1	Italy	2011	KY930929	KX520665
42	IBDV-HeN20-7103	A1bB1	China	2020	MW682877	MW682878
43	Irwin Moulthrop	A1aB1	USA	1967	AY029164	AY029165
44	ks	A3B2	Israel	1990	DQ927042	DQ927043
45	li4129/2014	A3B4	Finland	2014	MG739298	MG739299
46	Lukert	A1aB1	USA	1973	AY918948	AY918947
47	OH	A0B1	USA	1981	U30818	U30819
48	OKYM	A3B2	Japan	1991	D49706	D49707
49	P2	A1bB1	Germany	1977	X84034	X84035
50	P3009	A1aB1	Taiwan	1988	MH816964	MK040596
51	QZ191002	A2B1	China	2019	MZ066613	MZ066615
52	SH95	A3B1	China	1995	AY134874	AY134875
53	SHG19	A2B1	China	2018	MN393076	MN393077
54	SHG352	A2B1	China	2018	MT179720	MT179722
55	STC	A1a	USA	1967	D00499	-
56	TASIK	A3B2	Indonesia	1994	AF322444	AF322445
57	TL2004	A1bB2	China	2004	DQ088175	DQ118374
58	UK661	A3B2	UK	1994	NC004178	NC004179
59	UPM1432/2019	A2B1	Malaysia	2019	MT505343	MT505348
60	variant E	A2B1	USA	1985	AF133904	AF133905

## Data Availability

The complete genome sequences generated in this study were submitted to the GenBank database (https://www.ncbi.nlm.nih.gov/genbank/; accessed on 16 August 2022) under accession numbers OP225970 and OP225971.
